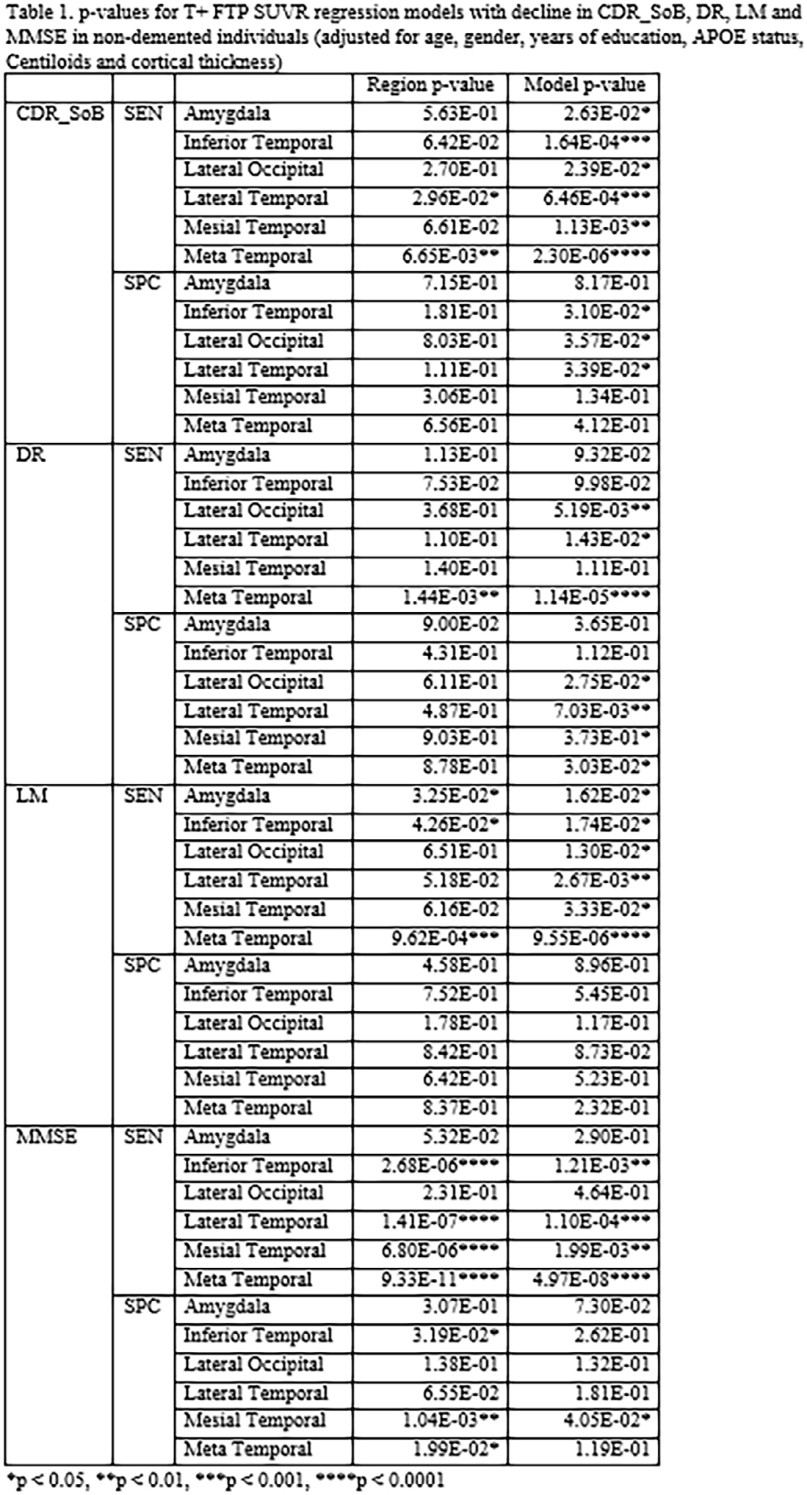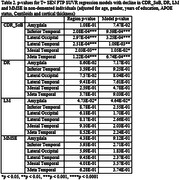# Assessing the efficacy of BETTH‐derived tau thresholds to predict cognitive decline in non‐demented individuals

**DOI:** 10.1002/alz.093128

**Published:** 2025-01-09

**Authors:** Alexandra Gogola, Brian J Lopresti, Beth E. Snitz, Dana Tudorascu, Davneet S Minhas, Vincent Dore, Milos D Ikonomovic, C. Elizabeth Shaaban, Julia Kofler, Cristy Matan, Pierrick Bourgeat, Neale S Mason, Christopher C. Rowe, Howard J Aizenstein, Chester Mathis, William E Klunk, Oscar L. Lopez, Ann D Cohen, Victor L Villemagne

**Affiliations:** ^1^ University of Pittsburgh, Pittsburgh, PA USA; ^2^ CSIRO, Brisbane, QLD Australia; ^3^ Austin Health, Melbourne, VIC Australia; ^4^ VA Pittsburgh Healthcare System, Pittsburgh, PA USA

## Abstract

**Background:**

Tau PET thresholds should detect early tau deposition and predict cognitive decline. We evaluated the relationships between the BETTH‐derived sensitivity and specificity tau thresholds (Gogola et al, doi:10.2967/jnumed.123.265941, 2023) and cognitive changes in non‐demented participants.

**Method:**

FTP scans from 314 non‐demented participants (age 73.17.1 years) and MK scans from 91 non‐demented participants (age 73.65.6 years) were processed and sampled to obtain regional SUVR values. Three cognitive measures were assessed: Clinical Dementia Rating Scale Sum of Boxes (CDR_SoB), Delayed Recall (DR), Logical Memory (LM), and Mini Mental State Examination (MMSE). Cognitive measures collected at the time of scanning were considered timepoint 0 (T0) and the most recently collected measures were considered timepoint 1 (T1). To evaluate the thresholds, SUVRs were split into below (T‐) and above (T+) threshold groups before assessment using both sensitivity (SEN) and specificity thresholds (SPC) for the FTP‐scanned participants and SEN thresholds for MK‐scanned participants, due to the limited number. Regression models between SUVR and change in cognitive measure, adjusted for age, gender, years of education, APOE status, Centiloids, cortical thickness and T0 cognitive score, were evaluated.

**Result:**

The FTP and MK regression model regional and full‐model p‐values are shown in Tables 1 and 2. For FTP, the SEN thresholds yielded greater levels of significance than the SPC thresholds. Additionally, for FTP, the Meta Temporal region was the most significantly associated with cognitive decline in all four metrics (p<0.0001). For MK, Meta Temporal, Inferior Temporal, and Lateral Occipital were significantly associated with CDR_SoB (p<0.001) and Amygdala was significantly associated with LM (p<0.05). No other associations were significant.

**Conclusion:**

The superior performance of the Meta Temporal region, for FTP, in all four cognitive measures indicates that cognitive decline is associated with cortical tau deposition. As seen with the MK results, this is particularly true as characterized by CDR_SoB. Moreover, the SEN thresholds were the best overall predictor of cognitive measure change, suggesting that they are the optimal regions and thresholds to apply in clinical diagnosis and therapeutic trials. Further work will assess the Meta Temporal SEN thresholds in relation to cognitive decline within the AT(N) framework.